# A Review on Current Designation of Metallic Nanocomposite Hydrogel in Biomedical Applications

**DOI:** 10.3390/nano12101629

**Published:** 2022-05-10

**Authors:** Nur Syafiqah Farhanah Dzulkharnien, Rosiah Rohani

**Affiliations:** 1 Department of Chemical & Process Engineering, Faculty of Engineering and Built Environment, Universiti Kebangsaan Malaysia, UKM, Bangi 43600, Selangor, Malaysia; p115201@siswa.ukm.edu.my; 2 Research Centre for Sustainable Process Technology, Faculty of Engineering & Built Environment, Universiti Kebangsaan Malaysia, UKM, Bangi 43600, Selangor, Malaysia

**Keywords:** metallic, nanocomposite, hydrogel, nanotechnology, drug delivery, tissue engineering, wound care

## Abstract

In the past few decades, nanotechnology has been receiving significant attention globally and is being continuously developed in various innovations for diverse applications, such as tissue engineering, biotechnology, biomedicine, textile, and food technology. Nanotechnological materials reportedly lack cell-interactive properties and are easily degraded into unfavourable products due to the presence of synthetic polymers in their structures. This is a major drawback of nanomaterials and is a cause of concern in the biomedicine field. Meanwhile, particulate systems, such as metallic nanoparticles (NPs), have captured the interest of the medical field due to their potential to inhibit the growth of microorganisms (bacteria, fungi, and viruses). Lately, researchers have shown a great interest in hydrogels in the biomedicine field due to their ability to retain and release drugs as well as to offer a moist environment. Hence, the development and innovation of hydrogel-incorporated metallic NPs from natural sources has become one of the alternative pathways for elevating the efficiency of therapeutic systems to make them highly effective and with fewer undesirable side effects. The objective of this review article is to provide insights into the latest fabricated metallic nanocomposite hydrogels and their current applications in the biomedicine field using nanotechnology and to discuss the limitations of this technology for future exploration. This article gives an overview of recent metallic nanocomposite hydrogels fabricated from bioresources, and it reviews their antimicrobial activities in facilitating the demands for their application in biomedicine. The work underlines the fabrication of various metallic nanocomposite hydrogels through the utilization of natural sources in the production of biomedical innovations, including wound healing treatment, drug delivery, scaffolds, etc. The potential of these nanocomposites in relation to their mechanical strength, antimicrobial activities, cytotoxicity, and optical properties has brought this technology into a new dimension in the biomedicine field. Finally, the limitations of metallic nanocomposite hydrogels in terms of their methods of synthesis, properties, and outlook for biomedical applications are further discussed.

## 1. Introduction

**Figure 1:**
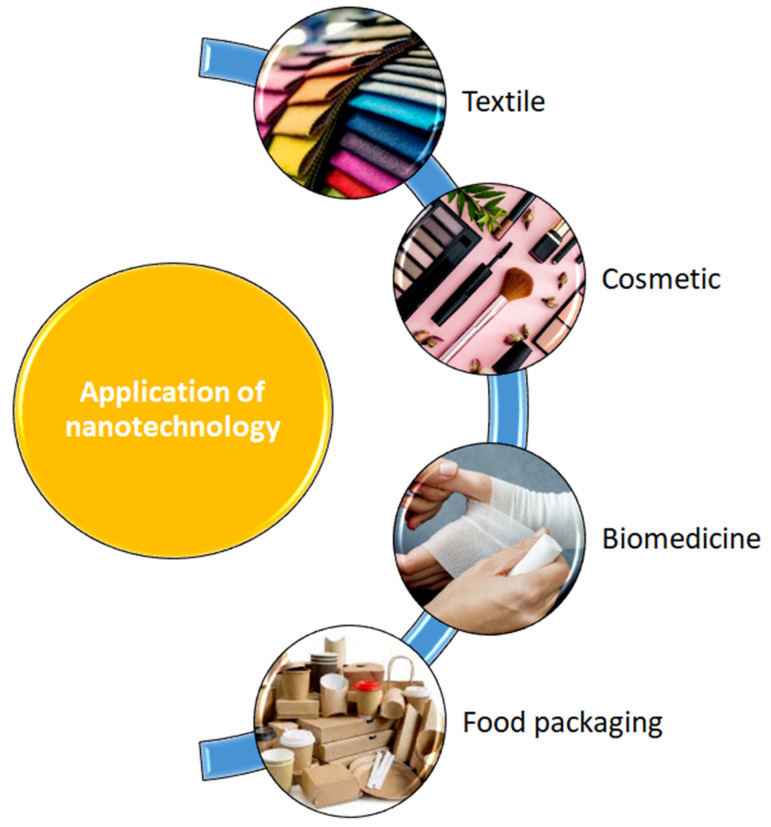
Involvement of nanotechnology in various industrial fields.

Nanotechnology is one of the latest fields in science and engineering; it deals with the design of nanoscale devices and systems, where the dimensions of the particles are less than 100 nm [1]. Since the 20th century, the advancement of nanotechnology through exploration and evolution has driven it to become one of the crucial domains for closing the gaps, both in the research and the industrial fields. The significant properties, such as the antimicrobial and electrocatalytic properties, thermal stability, luminescence, etc., can be transformed by nanotechnology and will eventually be applied to manifold applications, such as tissue engineering, biotechnology, biomedicine [2], cosmetics [3], food [4], textiles [5,6], and others. Some of the applications of nanotechnology in various fields are illustrated in Figure 1.

An extensive investigation of the drug delivery systems aided by nanotechnology has revealed excellent site-specific delivery and drug release properties to control targeted diseases. Over the past 2 years, the severe acute respiratory syndrome-associated coronavirus (SARS-CoV-2) pandemic has overwhelmed global healthcare systems. Thus, it is crucial that a rapid, early, accurate, cost-effective, and on-site diagnosis be invented to accommodate the spread of such viral diseases, including via nanotechnology. Ibrahim et al. [7] reviewed the effectiveness of gold (Au) and silver nanoparticles (Ag NPs) as biosensors in the detection of pathogenic RNA viruses. According to them, these NPs possess excellent characteristics, such as good biocompatibility, broad structural variety, and notable bio-imitative behaviours, for the efficient detection of pathogens.

Meanwhile, at present, the utilization of NPs can lead to unfavourable and longer-term side effects [8,9]. There is insufficient understanding of the particle size, morphology, absorption by immune system cells, safety risks, and toxicity, which are major concerns during the uptake of drugs [10]. Meanwhile, in the field of tissue engineering, despite the ability of nanocomposite materials to minimize the effects of tissue injury and artificial organ tissue, the fundamental knowledge about bio-mimic materials incorporated with nanotechnology is still lacking in this area, and thorough investigative studies supported by clinical approval are required [11]. Although biomedicine nanotechnology has brought the healthcare sector to a new level, challenges in the design of these nanomaterials, such as poor reproducibility, specificity, efficacy, affordability, and adverse side effects, still need to be diligently overcome. In view of this, the present review article selectively focuses on the evolution of metallic nanocomposite hydrogels in recent years and their applications in the field of biomedicine, specifically in drug delivery, wound care, and tissue engineering.

A lot of research work has been carried out to extensively explore the fabrication of nanomaterials for use in the field of biomedicine [10,11]. These nanomaterials, which comprise selected materials integrated with NPs, are produced by various techniques, including both physical and chemical methods, as well as by considering the correlation between their particle size and morphology through either in vivo or in vitro studies. The fabrication process involves the selection of suitable techniques and materials for the synthesis of the nanomaterials. Therefore, the next section will further discuss the types and methods of the synthesis of NPs to gain a comprehensive understanding of the reported techniques being used to fabricate NPs.

## 2. Types and Methods of Synthesis of Nanoparticles

Various nanostructures, such as micelles, liposomes, dendrimers, metallic NPs, and nano-coatings, have been utilized in the development of nanotechnology, especially in the biomedicine field [12,13,14,15,16]. Among the various known NPs, metal or metal oxide NPs are the most promising ones due to their antibacterial properties in association with a high surface-to-volume ratio [17]. The potential of NPs as antibacterial agents is dependent on their size, structure, and morphological behaviour, which could lead to different applications in the biomedical field, such as drug delivery, wound healing, and tissue engineering. Arokiyaraj et al. (2017) [18] elucidated the finding that Ag NPs extracted from the *Rheum palmatum* root exhibited an average particle size of 121.5 nm, with a zone of inhibition of 13–15 mm against the *S. aureus* and *P. aeruginosa*, produced through the well diffusion method. Recently, Feroze et al. (2020) [19] reported that the average size of Ag NPs synthesized from fungal metabolites of *Penicillium oxalicum* ranged from 60 to 80 nm. Their results demonstrated a better antibacterial performance, with a maximum zone of inhibition of 17.5 ± 0.5 mm (against the *S. aureus* and *S. dysenteriae*) and 18.3 ± 0.60 mm (against the *S. typhi),* using a similar method. Both of the referred works signified that the antibacterial activities of NPs are directly correlated to the size of the NPs, where the smaller the size of the particles, the higher their surface-to-volume ratio, thereby leading to their better performance as antibacterial agents.

NPs are known as particulate dispersions or solid particles or groups of atoms with dimensions in the range of 1–100 nm. Alterations to the surface area and/or volume of the NPs will lead to modifications of their properties, such as their mechanical, thermal, optical, and catalytic behaviour, which can be exploited in diverse applications, such as in the biological fields [20,21]. In the last decade, the fabrication of NPs based on polysaccharides [22], polymers [23,24], and plant-derived bioactive compounds [25] incorporated with active drugs has proven to be efficient in treating various pathological conditions.

**Figure 2:**
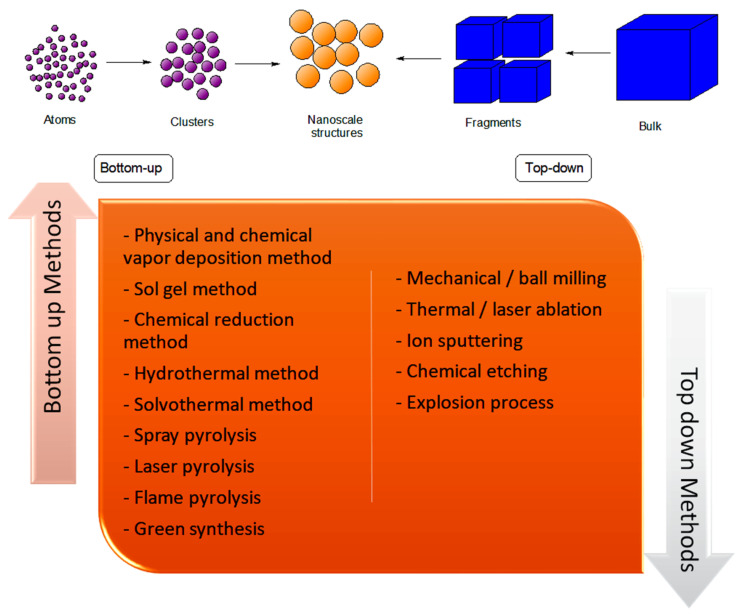
List of different bottom-up and top-down methods commonly used for NP synthesis.

The synthesis of NPs can be classified into two major methodologies, namely the bottom-up and top-down methods. Generally, in the bottom-up approach, atoms, molecules, or small particles are joined together to form a nanostructured building block of NPs. A few examples of the use of the bottom-up approach in the preparation of NPs are physical and chemical vapour deposition, liquid state synthesis, chemical reduction, gas phase, and the solvothermal methods [26]. In contrast, the synthesis of NPs using the top-down technique involves reducing the size of the bulk material via different physical and chemical treatments. Some of the physical techniques that are commonly used in the top-down method are lithography, laser ablation, sputtering deposition, pulsed electrochemical etching, and vapour deposition [27]. The techniques that have been discussed herein are illustrated in Figure 2.

**Figure 3:**
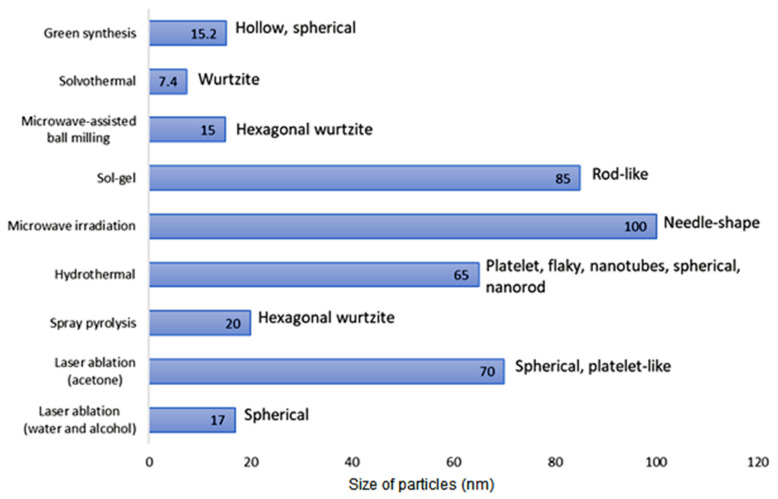
Comparison of the average size of particles (in nm) and morphological structures of ZnO NPs synthesized using different techniques: solvothermal [28], laser ablation [29], microwave irradiation [30], green synthesis [31], microwave-assisted ball milling [32], sol-gel [33], hydrothermal [34], and spray pyrolysis [35].

The utilization of different techniques and materials in the fabrication of NPs yields NPs with distinct properties, such as variations in the particle size and structure, morphology, and intrinsic behaviour. The bar chart in Figure 3 illustrates the average particle size of ZnO NPs synthesized using a variety of techniques. The chart indicates the different techniques and solvents used to obtain a distinct particulate size, eventually leading to a variety of significant properties in the ZnO NPs. Among all the techniques mentioned, the ZnO NPs produced by the solvothermal method exhibited a small particle size distribution, which was actually less than 10 nm. The use of ethanol as a solvent led to the formation of small, highly-dispersed ZnO NPs with a uniform shape and size [28]. This finding showed that the solvent plays an important role in the synthesis of ZnO NPs. A similar work by Thareja et al. (2007) [29] supported the use of water and alcohol as solvents to produce smaller-sized ZnO NPs (17 nm), which were significantly smaller than the size of the ZnO NPs (70 nm) produced through the use of acetone as the liquid media in the laser ablation method. In contrast, Hasanpoor et al. [30] described the fabrication of ZnO NPs using the microwave irradiation technique, where the nanoparticles displayed a particle size ranging from 50–150 nm and had a variety of morphologies at the different radiation powers of 540 W and 680 W. In fact, the morphological properties of the ZnO NPs also varied with the use of different techniques and solvents during the synthesis of the ZnO NPs, as presented in Figure 3. Therefore, the particle size and morphology of the NPs are dependent on the preparation technique and the solvent used for the construction of the NPs.

Nowadays, scientists have come to realize that most of the conventional methods (physical and chemical techniques) used for the synthesis of NPs can have major adverse effects on humans and nature. The physical methods used for the preparation of NPs include mechanical/ball milling, chemical etching and precipitation, microwave decomposition, thermal/laser ablation, and so on [36,37]. These methods come at a high cost in terms of resources, energy, time, and space and are considered as inconvenient for application in the research and industrial fields [38]. Meanwhile, common conventional methods that utilize chemicals, such as solvent extraction–evaporation, solvent diffusion, organic phase separation, sol gel, laser pyrolysis, and others, are usually associated with the utilization of hazardous organic solvents that are harmful to the environment and can affect the physiological structure [39]. In fact, these chemical methods can lead to the formation of toxic by-products and involve expensive, complicated, and laborious processes, which are unfavourable to the ecosystem, and lab work [40]. For that reason, it is necessary to use alternative synthesis pathways to eliminate most of the adverse effects, especially in an effort to minimize the disposal of waste and to avoid the possible use of hazardous and toxic materials. Despite the unique features of NPs that are beneficial for diverse applications, numerous nanomaterials have also exhibited toxicity at the nanoscale level. In order to eliminate this issue of toxicity, researchers and scientists have explored the fabrication of bio-nanoparticles by incorporating green chemistry into nanotechnology with the use of plants, microbes, etc. [41]. The green synthesis method has become a promising route to be explored by scientists as it uses biological sources, such as plant, bacteria, or fungi, to develop eco-friendly, non-hazardous, and biocompatible NPs. The plant-mediated green synthesis of NPs is a well-known method that utilizes plant extracts. This green synthesis method has gained significant attention among researchers exploring the different behaviours/properties of NPs in relation to the various type of plants used [42]. Sur et al. (2018) [43] utilized biosynthesized Ag NPs obtained from Reetha (*Sapindus mukorossi*) and Shikakai (*Acacia concinna*) plant extracts as an effective surface-enhanced Raman scattering (SERS) active substrate for the rapid identification of harmful bacteria, such as *Mycobacterium tuberculosis*. In another work, reported by da Silva et al. (2019) [44], a rare transition metal oxide of zirconia NPs (ZrO_2_ NPs) was successfully fabricated using *Euclea natalensis* plant extract as a bio-reducing agent. Recently, Abel et al. (2021) [45] reported on the green synthesis of ZnO NPs via an aqueous coffee leaf extract *(Coffee arabica)*, which served as a reducing agent to stabilize the particle length. This had a valuable antibacterial potency on pathogens in wounds. Meanwhile, Phang et al. (2021) [46] elucidated the potential of using CuO NPs from the water extracts of papaya (*Carica papaya* L.) peel biowaste as a photocatalyst for the degradation of palm oil mill effluent (POME) in wastewater treatment under ultraviolet (UV) irradiation. It was noticed that about 66% of the COD was reduced in the POME solution with the use of the biosynthesized CuO NPs under UV light irradiation for 3 h, indicating the degradation of soluble proteins and carbohydrates in the POME. The result showed that there was an improvement in the photocatalytic degradation of POME in the presence of the CuO NPs when compared to the absence of the NPs in dark conditions. Thus, the research work proved that NPs are considered as a promising photocatalyst in accelerating the treatment of POME wastewater.

Overall, physical and chemical methods have been developed to produce NPs. Nevertheless, these approaches may have a negative impact on the environment, human health, and the ecosystem. A few of the major concerns that have arisen in the production of NPs are to do with their high cost, hazardous nature, and the formation of toxic by-products. In order to overcome these issues, the development of an alternative route that is eco-friendly, affordable, and facilitates the creation a sustainable ecosystem is highly necessary. Green synthesis has become an alternative option and a promising pathway to replace the physical and chemical methods for the preparation of NPs as it is biocompatible and environmentally friendly. The rising popularity of the green synthesis method has triggered scientists to produce NPs from diverse sources, such as plant, bacteria, fungi, and algae. However, microorganisms, such as bacteria, fungi, and yeast, are exposed to the risk of culture contamination, tedious operations, and less control over the size of the NPs. Meanwhile, plant-mediated bio-synthesis has received wide attention for the fabrication of NPs due to the abundance of sources and the various choices of flora that can be explored. A review on the fabrication of metal or metal oxide NPs derived from plants will be presented in the following section.

## 3. Recent Advances on Plant-Mediated Nanoparticles

Recently, NPs synthesized from metals (i.e., Ag, gold (Au)) and metal oxides (i.e., ZnO, titanium oxide (TiO_2_), copper oxide (CuO), iron oxide (Fe_2_O_3_), and alumina (Al_2_O_3_)) have attracted tremendous attention in the research world due to their potential as strong antibacterial agents as a consequence of their large surface-area-to-volume ratio [47]. The introduction of transition materials into their structures makes them the best candidates for the synthesis of metal-based NPs as they possess partially filled d-orbitals in their elements, which makes them more redox-active (easier to reduce to zerovalent atoms), a feature that facilitates the aggregation of their NPs [48].

Usman et al. (2019) [49] prepared a series of gold NPs (Au NPs) at various concentrations of palm oil leaf extract via the ultrasound radiation technique. They confirmed that increasing the concentration of the palm oil leaf extract (POLE) during synthesis resulted in an increase in the average particle size and a decrease in the polydispersity index (PDI) of the synthesized Au NPs. In another work, by Ramimoghadam et al. (2013) [50], ZnO NPs were successfully synthesized using palm olein (PO) as a bio template via the hydrothermal method. Their findings showed that the particle size distribution tended to exhibit a dual mesoporous distribution, namely micro- and nano-structures with various volumes of PO. Ismail et al. (2019) [51] also worked on the effect of using honey on copper nanoparticles (Cu-NPs) via a green method aided by ultrasonic irradiation. An evaluation of the antibacterial assay and its cytotoxicity against selected bacteria and mammalian cell lines, respectively, revealed that the Cu NPs without honey had a higher cytotoxicity and higher killing activity compared to Cu NPs with honey. A study by Sharma and co-workers (2020) [38] reported on the potential photocatalytic and antimicrobial activities of synthesized ZnO NPs via the green synthesis technique. This study further revealed that the largest zone of inhibition was predominantly attained by *B. subtilis*, followed by *E. coli* and *S. aureus*. Furthermore, the minimum inhibitory concentration (MIC) and minimum bactericidal concentration (MBC) values were between 195 and 3125 μg mL^−1^ and 6250 and 12,500 μg mol^−1^, respectively, with these outcomes being lower than the values reported in previous findings [52,53], where the chemical-based approach had been chosen as the method of synthesis. Eventually, the ZnO NPs synthesized via the green method clearly showed that the plant-mediated ZnO NPs were more efficient as antibacterial agents. Table 1 shows a list of the NPs fabricated via green synthesis over the past five years (2018–2022). According to the table, a variety of metal or metal oxide NPs from various plant sources are considered as potential antimicrobial agents that can kill or inhibit the growth of various microorganisms. It is believed that these plant-mediated NPs can be utilized in diverse applications and industries where there is a high risk of contamination by bacteria and fungi.

Nevertheless, most of the metal and metal oxide NPs, such as ZnO NPs, Ag NPs, TiO_2_ NPs, and others, are highly toxic to human health and have become a major concern with regard to clinical and pharmaceutical handling. Cytotoxicity, oxidative stress, and mitochondrial dysfunction can occur in various cell lines due to the reduction in Zn-dependent enzymes and transcription factors by an increase in [Zn^2+^] ions, as a result of the dissolution of ZnO NPs in the extracellular region [65,66]. This was supported by Chen et al. (2019) [67], who stated that the cytotoxicity of ZnO NPs in HepG2 cells is dependent on the size or concentration of the ZnO NPs. According to Aadli et al. (2019) [68], lignin-functionalised Ag NPs have a toxic effect on MCF-7 cancer cells in a dose-dependent manner, and they suggested that the cell toxicity of Ag NPs might possibly be due to the generation of reactive oxygen species (ROS), such as hydrogen peroxide or their conversion into highly reactive hydroxyls or superoxide radicals. Niska et al. (2018) [69] mentioned in a review that TiO_2_ NPs also have a toxic effect, whereby the NPs accumulate on the surface of human keratinocytes (HaCaT cells), are absorbed by endocytosis, and generate ROS to cause mitochondrial damage to DNA and genotoxicity in human keratinocytes.

Thus, it is necessary to select a suitable method and the raw materials for a proper design of nanomaterials to reduce the toxicity of the substances. One such potential method involves reducing the dissolution of the toxic particulates to eventually hinder or lessen the toxicity of the materials. This can be done by modifying the composition or by doping in an extra component to improve the stability of the NPs. In addition, the addition of exogenous chelating agents in the synthesis may help to reduce the toxicity of the dissolved ions [70]. Díez-Pascual et al. (2015) [71] investigated the wound healing potential of polymeric films reinforced with chitosan-modified ZnO NPs and suggested that the modification of the ZnO NPs with chitosan reduced their cytotoxicity towards human cells. This was attributed to the increase in size of the NPs due to the physical attachment of biopolymers to the ZnO surface. Norouzi et al. (2021) [72] reported an insignificant loss of cell viability in HFF cells at a concentration of less than 500 μg/mL of polyvinyl alcohol/ZnO NPs (PVA/ZnO) through a cytotoxicity assay. The result showed that at concentrations below 500 μg/mL, the cell viability of HFF cells was higher than 82.8%, thereby indicating that PVA/ZnO NPs are non-toxic to human cells at that concentration level. The findings on the cytotoxicity of ZnO NPs from the above works were compared with the reported works [73], and it was revealed that there was an improvement in the toxicity of NPs on human cells when PVA was integrated into the NPs.

Recently, hybrid hydrogels together with nanotechnology have shown a significant potential to be used as antimicrobial agents. Some of the latest research work related to nanocomposites that has been reported in the open literature will be discussed in the subsequent subtopic.

## 4. Innovation of Metallic Nanocomposite Hydrogels

Recently, hydrogels have been attracting much attention among scientists due to their fascinating ability to absorb and retain a sufficient amount of water. A hydrogel comprises a network of monomers or polymers linked together by hydrogen bonds, electrostatic interactions, hydrophobic interactions, or covalent bonds to form a hydrophilic 3-D polymer network [74]. The medical application of nanotechnology, which is recognized in an emerging field known as nano-medicine, is still under development.

**Figure 4:**
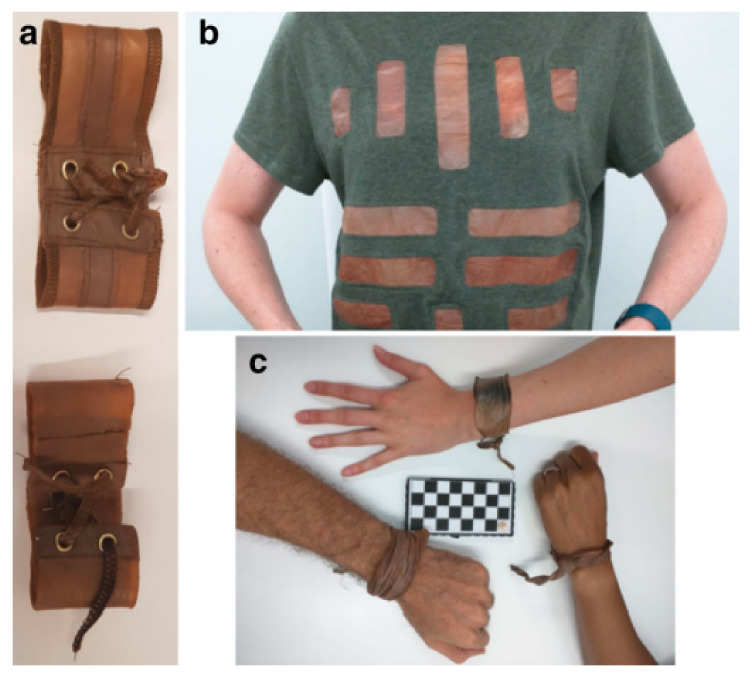
Photographs of (**a**) wristband, (**b**) T-shirt sewn with fabric coated HGBC, (**c**) condition of wristband after 2 weeks. Adapted from Ref. [77].

Farjadian et al. (2019) [75] evaluated the thermo- and pH-responsive characteristics of nano-carrier drug delivery platforms based on lysine-modified poly (vinylcaprolactam) (PVCL) crosslinked by poly (ethylene glycol) diacrylate (PEGDA) to form a nano-hydrogel conjugated with doxorubicin (DOX). The release profile of PVCL-DOX revealed that the highest release of 80% was obtained at 40 °C and pH 5 within 72 h, which indicates that the substance can suitably adhere to cancer sites. Additionally, PVCL-DOX showed an efficient response in killing cancer cells in in vitro studies on MCF-7 cell lines compared to that of the free DOX. This indicates that the prepared substance has a potential to be utilized as an effective anti-cancer agent. Meanwhile, Abbaszadeh et al. (2020) [76] fabricated a novel chitosan-based quercetin nanohydrogel (ChiNH/Q) and highlighted that the nanocomposite had distinct antitumour and anti-inflammatory properties that could regulate the proliferation of breast cancer cells. This was a novel finding as it demonstrated, for the first time, the application of ChiNH/Q on the global genomic DNA methylation profile of HEPG2 cells. A study into the antitumour properties of the prepared ChiNH/Q revealed that the level of methylated cytosine was significantly higher (1.01%) than the free-Q and ChiNH-treated cells (0.993% and 0.992%, respectively). This demonstrated the efficiency of this nanohydrogel in inhibiting tumour cells by decreasing the DNA methyltransferases (DNMTs) gene expression and also by increasing the global DNA methylation in HepG2 cancer cells. In another work, Kamiński et al. (2020) [77] investigated the potential behaviour of the synthesized polymer hydrogel, HydroGel Bacterial Cellulose (HGBC), which originated from a kombucha-derivative, as a replacement for synthetic materials, such as polyamide (nylon), polyester, acrylic, Kevlar, etc., in the textile industry, especially for use by astronauts in space. This HGBC material is one of the future innovations that is being explored widely due to its excellent mechanical durability, good swelling properties, fire-resistance, and comfort. Figure 4 shows photographs of the finished materials using HGBC for clothing.

Nevertheless, one drawback of using hydrogels on their own is their low mechanical strength, especially when used as scaffolds, which require high mechanical strength with good tolerance of compression and elasticity. This low mechanical property hinders their utilization for handling and loading in different parts of the body. Thus, the insertion of metallic NPs into hydrogels to form metallic nanocomposite hydrogels can increase the entanglements in its polymeric matrix to yield a high mechanical strength [78]. Dil et al. (2018) [79] reported on the antibacterial potential of novel porous gelatine silver/AcA (NPGESNC-AcA) nanocomposite hydrogels through free radical polymerization. According to their study, the gelation hydrogel with Ag NPs gave a better performance against *E. coli* (Gram-negative bacteria) compared to *S. aureus* (Gram-positive bacteria). This result may be due to the fact that the cell walls of Gram-negative bacteria have a thinner lipid layer that could have facilitated the penetration of the released Ag NPs into the bacterial cell membrane to eventually destroy them. Gholamali et al. (2019) [80] developed a new drug delivery system comprising oxidized starch/CuO nanocomposite hydrogels through the in situ method. They performed in vitro drug release profiles at different concentrations of CuO NPs on the hydrogels to determine the cumulative release of the drug from the metallic nanocomposite hydrogels. The reduced cumulative release of the drug, ibuprofen, with respect to increases in the concentration of NPs in an acidic environment was attributed to the role played by the CuO NPs as the primary precursor of the prolonged drug release behaviour. The outcome showed that a lower concentration of CuO NPs in the hydrogel matrix provided a better drug release profile, making it suitable for controlled drug delivery. In another work, Ahmadian et al. (2019) [81] also obtained a similar outcome with regard to the drug delivery potential of polyvinyl alcohol/CuO (PVA/CuO) nanocomposite hydrogels at pH 7.4. It was observed that as the number of CuO NPs increased, the drug release rate was lowered because the entrapped drug molecules could not easily diffuse into the contacted hydrogel network. Katas et al. (2021) [82] successfully synthesized Ag NPs using a spent mushroom substrate (SMS) as a reducing agent. The NPs were then incorporated with genipin-crosslinked gelatine hydrogels as a wound dressing. The biosynthesized AgNP hydrogel proved to be an effective antibacterial material against *S. aureus*, *P. aeruginosa*, *Bacillus subtilis* (*B. subtilis*), and *Escherichia coli* (*E. coli*).

Concurrently, any change in the size of the NPs is expected to alter their physical and chemical properties due to an increase or decrease in their surface area. Meanwhile, the hydrophilic properties and high-water content of hydrogels could cause outstanding mechanical and swelling properties. Thus, combining NPs and hydrogels under the same mechanism is a technological development that will result in nanocomposites with outstanding properties for applications in drug delivery, wound dressing, agriculture, textiles, and others. The fabrication of nanocomposite hydrogels incorporated with metallic NPs is still new, and thus, comprehensive and extensive studies are essential to obtain a full picture of the functionality of these types of nanocomposites in various applications. In this review, the focus on the utilization of metallic nanocomposite hydrogels in the field of biomedicine will be discussed in the next subtopic.

## 5. Functionalization of Biobased Metallic Nanocomposite Hydrogels in Biomedicine Applications

### 5.1. Drug Delivery Systems

It has been ascertained worldwide that the use of NPs for drug delivery involves complex research, with promising outcomes being generated from in vitro studies and small animal models. Nevertheless, research into the use of these nanomaterials on humans is still limited due to the translational gap that exists between animal and human studies. It has been observed that the behaviour and functionality of nanomedicines in the body are associated with the physiological and pathological differences between animal model species and humans [83].

Nanotechnology is a novel solution for overcoming the bottleneck in the issue of complexity owing to the fast development of nanoscience and the magnificent performance of nanomaterials. A nano-drug delivery system comprises nanomaterials with the full potential to enhance the stability and water solubility of drugs, extend the cycle time, elevate the uptake rate of target cells or tissues, and reduce enzyme degradation, thereby improving the safety and effectiveness of the drugs [84]. Two main aspects in the fabrication of metallic nanocomposite hydrogels are their pore size and their water intake, which must be taken into account during the drug-loading process to achieve the optimum release rate of the drugs into the targeted cells. The pore size of the nanocomposite hydrogel must fit the size of the immobilized drugs, while a high-water content is necessary to create a convenient environment for the immobilization of the drugs [85].

**Figure 5:**
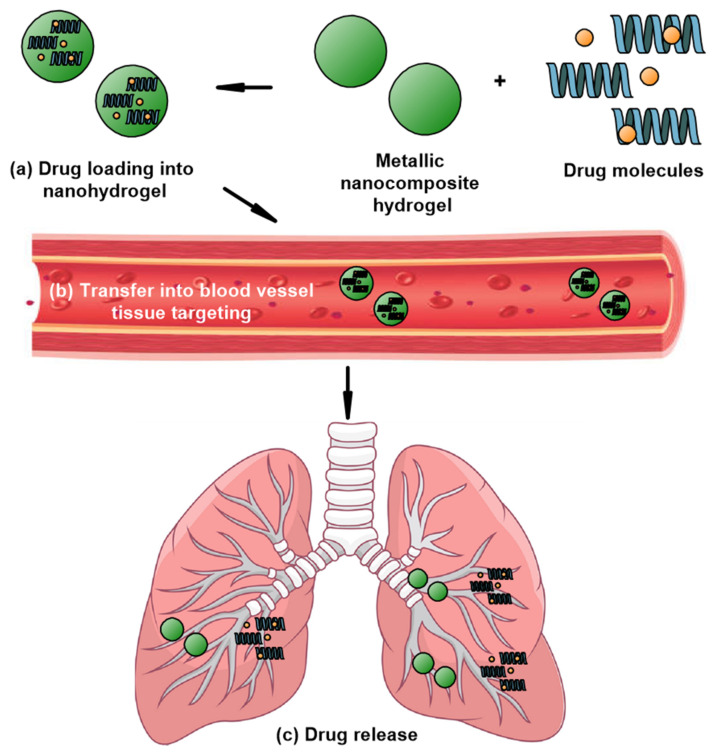
The proposed schematic diagram showing stages of drug delivery mediated by metallic nanocomposite hydrogel: (**a**) drug loading into metallic nanocomposite hydrogel, (**b**) immobilization of metallic nanocomposite hydrogel in blood vessel to targeted tissues, and (**c**) drug release process.

The schematic diagram in Figure 5 illustrates the drug delivery mechanism. In brief, Figure 5a shows that the drugs, made up of polymers, are loaded into the metallic nanocomposite hydrogel through physical or chemical attachments [86]. Next, this nanomaterial is administered into the patient’s body via an injection and is then transported into the blood vessels, as depicted in Figure 5b. At a certain point, due to the different environments at the site of the tumour, such as the presence of enzymes or a difference in pH, temperature, electric field, and redox potential, this condition could trigger the smart nanomaterial to release the drug loaded in the nanocomposite to interact with the tumour cells. The drug/therapeutic agent can be released from the nanocomposite hydrogel through three mechanisms, namely (i) diffusion-controlled or (ii) chemically controlled degradation or (iii) swelling-controlled mechanisms. As shown in Figure 5c, the immobilization of these drugs in the targeted tissues can cause the partial or complete destruction of tumour cells [87,88].

**Figure 6:**
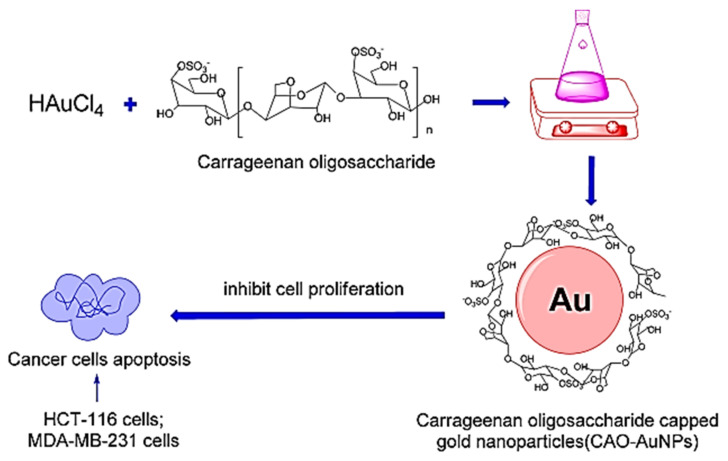
Schematic diagram showing the mechanism of carrageenan oligosaccharide (CAO) hydrogel capped the gold nanoparticles (Au-NPs). Subsequently, the cytotoxicity test of CAO-AuNPs nanocomposite hydrogel on cancer cells (HCT-116 cells and MDA-MB-231 cells) was also conducted. Adapted from Ref. [91].

Amiri et al. (2017) [89] successfully prepared novel alginate/CoFe_2_O_3_ metallic nanocomposite beads (Alg/CFO MNPs) hydrogels in the presence of caffeine via the co-precipitation method. This work is claimed to be the first to deal with a combination of alginate hydrogel beads with magnetite cobalt ferrite nanoparticles (CFO MNPs) as a drug carrier. The in vitro study proved that the release profile of this nanocomposite hydrogel was elevated at neutral pH compared to an acidic environment, which influenced the diffusion of drugs into the nanomaterials. In an acidic environment, the hydrogels are mainly surrounded by carboxylic acid and easily undergo protonation, which eventually results in a decline in drug diffusion. Basu and co-workers (2018) [90] developed semi-interpenetrating-hydrogel-silver nanocomposites (SNA) via a facile and simple green methodology through the free radical copolymerization of sodium alginate/polyacrylamide (NaAlg/PAAm) onto *Dolichos biflorus* Linn, which acted as a reducing agent. According to their study, the loading efficiency of the drug was greatly induced by the concentration of the drug and the impregnation time. Both factors contributed to the high increase in the drug loading in the initial phase, which eventually dropped off at a certain concentration and time. These effects may be attributed to the high drug diffusion level during the early loading stage. Meanwhile, Chen et al. (2018) [91] successfully fabricated Au NPS from carrageenan oligosaccharide (CAO) hydrogels, which were used as the reducing and capping agent. They investigated the antitumour activities of their synthesized NPs on colorectal cancer and breast cancer in vitro. Based on the synthetic route and mechanism shown in Figure 6, there was a reduction of Au^3+^ to Au^0^ ions in the reaction system, indicating the formation of Au NPs. Meanwhile, the CAO, which acted as a capping agent, was added to obtain CAO-Au-NPs nanocomposite hydrogels. The CAO was surrounded by gold atoms to form nano-sized particles.

Organic–inorganic hybrid hydrogels based on an interpenetrating polymer network (IPN) synthesized from polyaspartic-acid-based polymers embedded with Ag, CuO, and ZnO NPs were successfully designed by Sattari et al. (2018) [92]. In general, the fusion of NPs and IPN hydrogels increased the stability of the drug, curcumin, in the network and eventually caused a slower release of the drug. In addition, Sattari and co-workers also concluded that the pH environment affected the release of curcumin from the synthesized hydrogel, where it was observed that there was an increased release of the drug from the hydrogel at its natural pH due to an increase in the electrostatic repulsion and a reduction of the hydrogen bonds between the ionic groups. Recently, Nagaraja et al. [93] elaborated on the potential use of natural polymer-based stimuli-responsive silver nanocomposite hydrogels (TGIAVE-Ag) obtained through a simple redox polymerization and a green method using an aqueous extract of *Terminalia belliricia* seeds. The prepared TGIAVE-Ag exhibited excellent antimicrobial activity with a large inhibition zone ranging between 12–18 mm against *K. pneumonia, P. aeruginosa, E. coli*, and *S. aureus* as Gram-positive and Gram-negative bacteria, through the disc diffusion method. They suggested that the Ag NPs generated free radicals and Ag^+^ ions, which led to the formation of pits and the eventual death of the cells.

In general, natural sources are used to synthesize green NPs, which act as a reducing and stabilizing agent, before the metal atoms are reduced to metal ions. These nano-scale particles will eventually combine with the hydrophilic hydrogels, which commonly contain –OH, –COOH, –CONH_2_, –CONH, and –SO_3_H, to elevate the rate of drug diffusion into the targeted cells. Tumour cells or tissues usually behave differently in the environment compared to normal cells, and therefore, the materials must be able to withstand the changes in the behaviour of tumour cells while transporting the drugs. Metallic nanocomposite hydrogels have shown their ability to adapt to external stimuli, such as pH, temperature, and enzymes, at the tumour site, thus enhancing the diffusion rate of the therapeutic agent in the drug delivery system. In addition to drug delivery, these nanomaterials have also exhibited a potential in wound care applications, which will be discussed in the following subsection.

### 5.2. Wound Care Applications

Skin is the largest organ of the body and comprises multiple components and functions. The outer layer of the skin is composed of dead cells and the epidermis, which acts as a protection against the environment. The dermis or so-called middle layer, which consists of living cells with a network of blood vessels and nerves, is responsible for the detection of any external stimulus and the thermal regulation of the enclosed body. The inner layer of the skin is mainly formed of fat, and its function is to insulate the body against shock. A wound is caused by a physical trauma, where the skin is torn, cut, or punctured (an open wound) or where a blunt force trauma causes a contusion (a closed wound) [94].

There are two types of wounds, namely acute wounds and chronic wounds. In general, acute wounds are injuries with minimal microbial load or zero infection, scab generation, and immune cell infiltration in the beginning stage. Consequently, acute wounds can be repaired within a short period of time [95]. On the other hand, chronic wounds take a longer time to repair due to the challenging process of being reimposed into the normal anatomical structure and functions [96].

**Figure 7:**
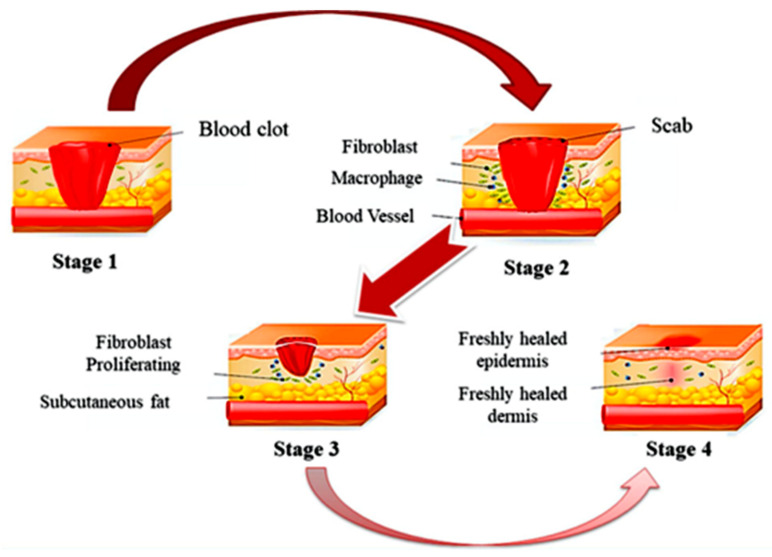
Illustration on the wound healing process. The four stages of wound healing, which are (i) Stage 1: Hemostasis phase; (ii) Stage 2: Inflammatory phase; (iii) Stage 3: Proliferation phase; and (iv) Stage 4: Remodeling phase. Reprinted with permission from Ref. [98]. Copyright 2020 Elsevier.

Wound healing is typically divided into four different phases, namely: (i) haemostasis, (ii) inflammation, (iii) proliferation, and (iv) remodelling [20]. Figure 7 shows a schematic diagram of the flow of the wound healing process. These phases are clarified in the following points:

(i) Stage 1: Haemostasis—once the skin experiences an injury, the haemostasis process will generate platelet clotting, accompanied by the formation of a fibrin matrix that acts as a scaffold for cell infiltration [97].
(ii) Stage 2: Inflammation—the second stage, which is the inflammation process, will start immediately after blood clotting, and it takes around 24 h to 4–6 days to complete the process. In this stage, proteolytic enzymes and pro-inflammatory cytokines will be secreted over the immune cells around the wound area. These inflammatory cells produce reactive oxygen species (ROS), which are responsible for protecting the organism from bacteria and infections. Gram-positive bacteria, such as *S. aureus* and *S. pyogenes,* can be found predominantly in the first stage of infection, while Gram-negative bacteria (i.e., *E. coli* and *P. aeruginosa*) dwell in wounds that have already developed. In this context, an extensive understanding of cell-material interactions is vital to trigger an exploration of new biomaterials. The discovery of new biomaterials, including nanocomposite hydrogels, which can mimic the microenvironment of the skin, is essential to enhance the rate of wound healing, especially in chronic diabetic patients. The metallic nanocomposite hydrogel should be able to target the macrophages to become regenerative and, eventually, be able to regulate the excretion of signalling molecules. Consequently, a polymeric matrix offers structural support and carries information to be passed to the cells for their in-growth.
(iii) Stage 3: Proliferation—all foreign particles and tissue debris are eliminated from the wound by neutrophils and macrophages, thus preventing infections [98]. The activation of the support system following the arrival of the neutrophils at the lesion is associated with the loss of granules in the platelet, the liberation of chemotactic signals by the necrotic tissue, and bacterial deterioration.
(iv) Stage 4: Remodelling—finally, the system will eventually evolve by causing the macrophages to react to injuries. These cells are in charge of the phagocytosis of fibrin and cellular debris, and they release the macrophage-derived growth factor (MDGF) for fibroblasts and endothelial cells [97].

The materials used to cover or treat wounds have also evolved from simple wound covers into commercial wound dressings, with the latest one involving smart technology. Originally, common materials, such as honey, animal oils or fats, cobwebs, mud, leaves, moss, or animal dung in a crude form were used as wound dressings, and later, two or more of these components were combined for convenience of use and to improve their clinical effectiveness. Up to the end of the 19th century, absorbent sheets were produced by scraping sheets of old linen with sharp knives. These were eventually replaced by cotton in the middle of the 20th century [94]. Wound dressings, such as gauze, sterilized absorbent cotton, and bandages, are commonly found in clinical practices due to their low cost. However, these dressings can only provide physical protection and are limited in their wound healing mechanisms when it comes to maintaining a moist environment and preventing infections [96]. Nowadays, smart wound dressing has emerged as a promising strategy for enhancing wound care management by modifying the materials to another level of innovation in advanced wound dressing technologies. The developed smart wound dressing is not only able to provide physical protection but can also act as a diagnostic sensor that is able to monitor the condition of a wound and apply a proper treatment to promote the healing process [99,100].

Recently, the emergence of metallic nanocomposite hydrogels in wound healing applications has driven these nanotechnologies into new inventions in the science and engineering fields. The role of these nanocomposite hydrogels in wound care is dominantly favourable due to their fascinating biocompatibility, controllable physical characteristics, natural drug-loading system, and abundance of functional groups [101]. A study by Haseeb and co-workers (2017) [102] revealed that their fabricated wound dressing containing silver nanoparticles (Ag NPs), produced from linseed hydrogels (LSH), showed excellent antimicrobial and wound dressing properties. Based on their antimicrobial activity, it was found that the LSH-Ag NPs actively inhibited the growth of several bacteria and fungi, such as the *S. mutans*, *S. epidermidis*, *P. aeruginosa*, *E. coli*, *S. aureus*, *B. Subtilis*, *A. odontolyticus,* and *A. niger* strains. Additionally, the LSH films impregnated with Ag NPs showed a wound closure of 100% for rabbits on the 15th day. A similar outcome was obtained from a commercialized Band-Aid dressing on the excised wound of a group of healthy male rabbits. Nevertheless, the excised wounds on a rabbit patched with the LSH-Ag NPs film displayed a faster recovery compared to the standard Band-Aid dressing.

Next, Ezealisiji et al. (2019) [103] reported on the successful synthesis of ZnO NPs nanocomposite hydrogels, using a *Solanum torvum* extract as the bio template, which were tested for their effects on the hepatic and renal functions of Wistar rats. They found that the moisturizing and humectant properties of the hydrogels enhanced the diffusion of the nanomaterials into the skin by reversibly agitating the stratum corneum, which eventually contributed to an added driving force for the transference of the nanomaterials into the skin. Later, the metallic nanocomposite hydrogels resulted in the initiation of oxidative stress, mitochondrial impairment, and DNA denaturation, while accelerating apoptosis of the skin structure. Another work by Batool et al. (2021) [55] revealed the effectiveness of ZnO-NPs/silica gel hydrogels (ZnO-NP/SG) fabricated from *Aloe barbadensis* leaf extract for wound healing in male albino mice. The prepared wound dressing showed a promising potential for the control of bacterial growth as it is biocompatible and can accelerate wound healing behaviours in mice, which proves its therapeutic ability. According to Batool et al., the high concentration of ZnO-NP/SG loaded at 30 ppm resulted in a higher wound recovery rate than in the control sample. Meanwhile, it was found that at a concentration of 15 ppm, it was capable of healing 95% of the wound within 11 days. These results showed that the ZnO-NP/SG dressing had a better control of bacterial growth, was biocompatible, and accelerated wound healing properties in mice, thereby indicating its therapeutic ability.

In summary, metallic nanocomposite hydrogels are considered as ideal materials for offering a protective barrier against bacterial infections, as well as in promoting the rate of wound healing, as they are biodegradable, biocompatible, non-toxic, antimicrobial, biologically adhesive, biologically active, and haemostatic. The moist condition of this nanomaterial is able to prevent wound dehydration, which could slow down the wound healing process. At the same time, the NPs embedded in the hydrogels are responsible for acting as an antimicrobial agent by inhibiting the formation and growth of bacterial or fungal strains. In fact, it has been proven in extensive research studies that nanocomposite hydrogels can replace the conventional Band-Aid dressings available in the market due to their potential to enhance the wound healing process at a faster rate than the standard Band-Aid dressings. With a deeper understanding and with further studies into nanocomposite hydrogels, these smart nanomaterials can be commercialized in the market in the future and can be safely used, either in daily life or in the clinical field. Next, the functionality of metallic nanocomposite hydrogels in tissue engineering applications will be explored and discussed in the subsequent sub-topic.

### 5.3. Tissue Engineering and Regenerative Medicine (TERM)

Many factors can contribute to the burden of orthopaedic trauma, and these mostly involve bone and cartilage fractures due to road accidents, work and sports injuries, degenerative diseases, such as osteoporosis, osteoarthritis, cancer, cystic fibrosis, and hereditary bone diseases, such as marrow abortion. The abrasion implications defined by bone defects, bone fractures, severe pain, fever, redness, and others demand a proper reformation and rehabilitation [104]. Moreover, various physiological signals, such as biochemical, mechanical, and electrical signals, are essential in the process of tissue regeneration. Tissue impairment normally produces local physiological electricity, followed by the interchanging transmission of electrical signals between cells. The emergence of external electrical signals in the tissue regeneration system can cause changes in the electrical charges between the cells and can stimulate the adherence, migration, proliferation, and differentiation of stem cells. Thus, it is essential to select suitable electrically active materials that are capable of transferring electrical signals through, for instance, a scaffold to build a biomimetic electro-micro-environment for the purpose of stem cell differentiation [105].

**Figure 8:**
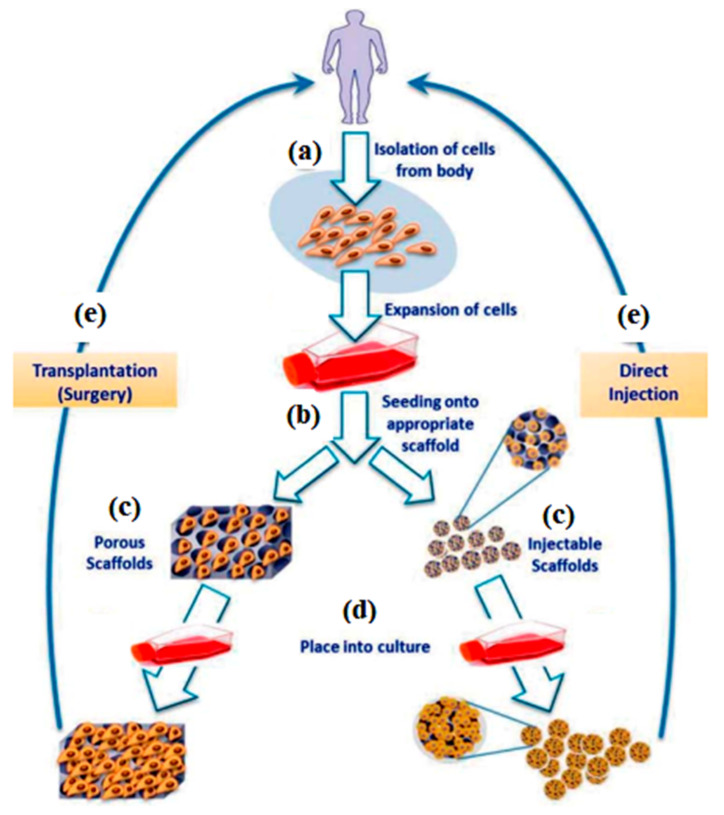
Schematic illustration demonstrated the most common tissue engineering mechanism, starting with (**a**) isolation of cells from body, (**b**) implantation cells onto scaffold, (**c**,**d**) cell proliferation, and (**e**) transplantation or injection. Adapted from Ref. [106].

In order to cater for injuries and diseases, the development of tissue engineering has shown a great potential for innovation in clinical applications. Tissue engineering and regenerative medicine (TERM) comprise a restoration process and the substitution or regeneration of damaged tissues, which are difficult to repair. Figure 8 presents the tissue engineering approaches that are commonly practised. Prior to the development of the tissue or organ, it is essential to isolate some cells from a small tissue biopsy performed on the patient to gain an understanding of the characteristics of the specific tissue cells, as depicted in Figure 8a. The isolated cells are then expanded and seeded into three-dimensional (3D) scaffolds that mimic the natural extracellular matrices (ECM) of the targeted tissue, where they undergo proliferation as well as differentiation, as shown in Figure 8b–d. The scaffolds are responsible for transporting the cells to the targeted site in the patient’s body, enhancing cell-biomaterial interactions, aiding cell adhesion, and enabling sufficient gases, nutrients, and growth factors to be transported to the seed cells to maintain the survival, proliferation, and differentiation of the cells. Subsequently, these cell-loaded scaffolds are transplanted into the patient by injecting or implanting the fabricated tissue at the desired site via surgery, as depicted in Figure 8e [106]. Thus, it is essential that the materials be fabricated according to the needs in the TERM applications by taking into account certain factors, such as the cell proliferation, the type of cells at the targeted site, and the signal connection.

Nowadays, the transplantation of tissues acquired from a healthy donor (an allograft) or from a patient’s own body (an autograft) is one of the many options available to patients. However, a few drawbacks that could occur during tissue transplantation are the lack of donor tissue, the possibility of infection, the high chance of tissue rejection, and faulty graft guts [107]. Over the past 5 years, numerous techniques, such as supercritical fluid technology and 3D printing, have been widely used to fashion the scaffolds for tissue engineering [108]. Hakimi et al. (2018) [109] invented a remarkable portable 3D bioprinter (weight < 0.8 g) with a microfluidic cartridge that can be utilized for the bioprinting of skin sheets. The study demonstrated a successful in situ bioprinting on porcine and murine wound models, where the wounds were fully covered with a homogeneous layer according to the thickness of the wound. The wounds treated with the in situ deposited sheet stopped bleeding approximately 5 min later and were able to achieve haemostasis after 10 min. Such portable 3D printers can be revolutionized in the current healthcare market, especially for emergency cases such as burn trauma that require immediate treatment [110].

Meanwhile, a metallic nanocomposite hydrogel possesses a network that acts as an artificial scaffold, which can be fitted into a 3D microenvironment. This approach has become an innovative technique in the modern TERM field, where the main goal of the TERM is to assemble functional constructs of tissues or organs by restoring and maintaining the damaged tissues or whole organs. In addition, this nanocomposite hydrogel has unique properties, such as high-water content, biocompatibility, stimuli-responsive features, and bio-responsive functions, which could offer various materials of choice for manifold TERM applications [111,112]. Although they possess water-bearing properties, the common hydrogel matrices are unable to conduct electrical signals and can obstruct the signal connection between cells. Nowadays, the development of conductive hydrogels offers a more promising potential in the tissue engineering field compared to the common hydrogels due to their capacity to stimulate electrical signals between cells and promote a physiological microenvironment for electroactive tissues [105].

Various research works have been established to create biomaterials that can mimic the microenvironment of the skin. Previously, Zulkifli et al. (2017) [113] reported on the fabrication of low-toxicity hydroxyethyl cellulose/silver NPs (HEC/Ag NPs) nanocomposite hydrogel scaffolds, which are ideal for skin tissue engineering applications. Rakhshaei et al. (2019) [114] prepared chitosan-gelatine/ZnO nanocomposite hydrogel scaffolds (CS-GEL/nZnO) through in situ synthesis and studied the cytocompatibility of the nanomaterial on normal human dermal fibroblast cells (HFF2). In comparison to the previous report [115], where the NPs were simply added to the matrix of the scaffold, the production of this nanomaterial through in situ synthesis was clearly able to elucidate a higher antibacterial and lower cytotoxicity effect because the ZnO NPs were well distributed in the polymeric matrix. Thus, the in situ synthesis of CS-GEL/nZnO hydrogels is strongly recommended for biomedical applications, especially for skin tissue engineering. Recently, Zhou et al. (2020) [111] reviewed the proliferation and expansion of cardiomyocytes using Au NPs fused with a silk-based hydrogel (SF/ECM). They reported that the uniform distribution of Au NPs in the matrix provided a favourable conductivity and had a biological effect on cardiac repair, where this metallic nanocomposite hydrogel was able to effectively decrease the size of infarct tissue from 89% to 65%.

In brief, TERM is one of the vital fields of biomedicine, and a detailed understanding of its mechanism and conceptualization is still required if it is to be applied in real-life applications. Although a hydrogel contains a certain amount of water and is biocompatible, the hydrogel itself possesses a low viscosity and is unable to conduct electrical signals, which makes it unfavourable for utilization in TERM. Thus, to satisfy the requirements for its use as a scaffold, the hydrogel matrix must be modified to obtain the required structure and properties. A few factors, such as cell proliferation, adhesion, migration, and signalling, must be considered in the engineered tissues prior to the fabrication of the metallic nanocomposite hydrogels to enable them to be used for the treatment of organ failures resulting from diseases and injuries. Combinations of the selected NPs and hydrogels can be tailored to create crosslinked networks with enhanced mechanical strength and electrical, optical, magnetic, and biomedical properties. In addition, nanocomposite hydrogels can be used as a temporary alternative to replace burnt or injured tissue as they have the ability to mimic the microenvironment of human tissue. However, inventions using this nanotechnology are still limited and must be further established in the future. Thus, the next section gives an overview of the limitations and future perspective of metallic nanocomposite hydrogel technology in order to identify important ideas that can be used to overcome the current limitations of this technology.

## 6. Limitations and Future Perspective of Metallic Nanocomposite Hydrogel Technology

In brief, the fabrication of metallic nanocomposite hydrogels containing NPs from various natural sources through green synthesis has shown a tremendous potential for diverse applications, especially in the biomedicine field. In previous studies in the literature [116,117], conventional hydrogels suffered from poor mechanical strength and fast/abrupt biodegradation limits, thus making them unfavourable for most applications [118]. Meanwhile, the instability of NPs in aqueous solutions has become a major constraint to the advancement of novel nanomaterials [119]. Therefore, various emerging technologies to reinforce hydrogels composed of nanomaterials are being identified to close the gap by enhancing the strength and elastic modulus of these nanocomposites. Although publications on NPs and hydrogels abound, publications on the fabrication of hydrogels incorporated with metallic NPs are still limited and need to be explored further. In the fabrication or modification of metallic nanocomposite hydrogels, a few factors, such as structural modification, material stability, processability, and solubility, which might affect the production, must be taken into consideration as these conditions will vary greatly and have an impact on the hydrogel cross-linking materials [120]. From another perspective, there are also concerns with regard to the adverse effects of the cytotoxicity of the nanomaterials used due to the controversial interaction between the uncertain mechanisms of the NPs and cells [121]. Therefore, the selection of the best materials and techniques for the preparation of these novel antimicrobial metallic nanocomposite hydrogels is crucial, and a few factors need to be taken into consideration in observing their biocompatibility with cell growth and their precise functions.

## 7. Conclusions

The introduction of biosynthesized metallic nanocomposite hydrogels has become a new alternative route for the replacement of non-biodegradable synthetic materials. The excellent properties of metallic nanocomposite hydrogels, such as their small size, moist condition, biodegradability, high thermal stability, conductivity, and good mechanical strength, have greatly affected the development of nanotechnology for various application fields, such as in biomedicine, coatings, food industry, packing, and others. In this review, a few of the current fabricated metallic nanocomposite hydrogels and their use in the field of biomedicine, such as in drug delivery, wound healing, and TERM, were discussed extensively. In addition to that, the limitations on the use of these metallic nanocomposite hydrogels were briefly explained at the end of this review. The most crucial challenge with regard to biosynthesized metallic nanocomposite hydrogels is that their complex mechanism and interaction with biomolecules have not been clearly distinguished. In addition, parameters such as the size, shape, agglomeration, purity, surface area, surface charge, functionalization, storage stability, and adverse cytotoxicity of the metallic nanocomposite hydrogels must also be considered during the synthesis of these nanocomposite. Overall, in-depth and comprehensive studies on the mechanism and compatibility of metallic nanocomposite hydrogels on human cells need to be carried out to create advanced smart wound dressings in the near future.

## Figures and Tables

**Table 1:** List of nanoparticles with their applications.

Nanoparticles	Technique	Pathogens	Application	Reference
CuO	Green synthesis (Aloe vera leaf extract)	*A. hydrophila, P. fluorescens, F. branchiophilum*	Fisheries	[54]
ZnO	Green synthesis *(Curcuma longa* L.)	*B. subtilis, K. pneumonia, B. licheniformis, E. coli, A. niger, C. albicans.*	Wound care	[55]
CuO	Green synthesis (*Carica papaya* leaves)	*B. subtilis, B. megaterium, B. cereus, S. epidermidis, E. coli, K. pneumonia, K. oxytoca, S. typhimurium.*	Textiles	[56]
Ag	Green synthesis (Mahua oil cake (MOC) of *Madhuca latifolia* L.)	*S. aureus, S. faecalis, L. monocytogenesi, E. coli, S. typhimurium*	Food packaging	[57]
Ag	Green synthesis (*Crocus sativus* L. wastages)	*E. coli, P. aeruginosa, K. pneumonia, S. flexneri, B. subtilis*	Biomedicine	[58]
Ag	Green synthesis (*Capsicum Annuum var Annuum*)	-	Tissue engineering	[59]
Au (gold)	Green synthesis (*Hubertia ambavilla*)	-	Cosmetics	[60]
Fe_3_O_4_	Green synthesis (*Lagenaria siceraria*)	*E. coli, S. aureus*	Drug delivery	[61]
Palladium (Pd)	Green synthesis (gum harri (*Anogeissus latifollia*)	*P. aeruginosa, S. aureus*	Catalyst	[62]
AgNO_3_, Cu(NO_3_)_2_, Ce(NO_3_)_3_, La(NO_3_)_3_, Zn(NO_3_)_2_	Green synthesis (*Senna occidentalis*)	*E. coli, S. aureus, C. globosum, A. alternata*	Paint coating	[63]
CdO	Green synthesis (green tea)	-	Electronic devices	[64]

## Data Availability

No new data were created or analyzed in this study. Data sharing is not applicable to this article.
